# Lectures on the Pathological Appearances Observed in the Bodies of the Insane. No. II

**Published:** 1850-07-01

**Authors:** John Hitchman

**Affiliations:** Resident Medical Officer to the Female Department of the Institution


### LECTURES ON THE PATHOLOGICAL APPEARANCES OBSERVED IN THE BODIES OF THE INSANE.

DELIVERED AT THE MIDDLESEX COUNTY LUNATIC ASYLUM,

BY DR. JOHN HITCHMAN,

*Resident Medical Officer to the Female Department of the Institution.*

NO. II.

IN my address on Saturday last, I endeavoured to give you some idea of the pathology of "General Paralysis." In this lecture, I shall have to solicit your attention to another complication of insanity, which unfortunately, like general paralysis, is in almost every case incurable; and I trust that that frightful word, "incurable," will stimulate your research, rather than diminish your interest in the disease. The complication to which I refer is "Mania with Epilepsy." If neither disease be well understood in its simple condition, we must expect obscurity in their compound form. Epilepsy is still a mystery. Notwithstanding the great labours of some modern pathologists, and the close observations of such able men as Marshall Hall, Todd, Wilson, and others, I fear we must confess that our knowledge of epilepsy is not much greater than that possessed by Aretæus, who flourished at Cappadocia during the reign of Vespasian. This is not because there have been no observers. Dr. Copland, in his great and marvellous work, the "Dictionary of Practical Medicine," gives references to more than two hundred writers on this subject. The disease has every attribute which is likely to arrest attention, and to excite research. It is sudden in its seizure, and hideous and appalling in its progress. It begins with a scream, which is the very accent of terror, and ends with a sleep, which sometimes merges into death. Its importance cannot be overrated. The very names which have been attached to it proclaim its moment. In the time of Hippocrates it was called "Morbus Sacer;" the elegant and classic Celsus styles it, "Morbus Major;" while Pliny and Seneca give to it the title of "Morbus Comitialis." It has been associated with the stars, and called "Astralis;" with the infernal regions, and styled "Scelestus." In one era, the unhappy patient has been deified; in another, and a later age, the convulsions have been regarded as the strugglings of a satanic power. Be it ours to regard it as a bodily disease, influenced by, and influencing the operations of, the mind. Adopting, in truth, the wise statement of Hippocrates, who says,—"Neither truly do I count it a worthy opinion to hold that the body of man is polluted by God, the most impure by the most holy; for were it deified, or did it suffer from any other thing, it would be like to be purified and sanctified, rather than polluted by God. . . . . . But this disease seems to me to be nowise more divine than others; but it has its nature such as other diseases have, and a cause whence it originates, and its nature and cause are divine only just as much as all others are; and it is curable no less than the others, unless when from length of time it is confirmed, and has become stronger than the remedies applied. Its origin is hereditary like that of other diseases."—(*The Sacred Disease. Sydenham Translation.* 1846.) The symptoms of epilepsy are familiar to you. The disease has been thus graphically portrayed by Lucretius:—

> "Quin etiam, subito, vi morbi sæpe coactus

> Ante oculos aliquis nostros, ut fulminis ictu,

> Concidit, et spumas agit; ingemit, et tremit artus;

> Desipit, extentat nervos, torquetur, anhelat

> Inconstanter, et in jactando membra fatigat.

> . . . . . . . . . .

> Inde ubi jam morbi reflexit caussa, . .

> . . . . . . . . . .

> Tum quasi vacillans, primum consurgit et omneis

> Paullatim redit in sensus, animamque receptat.

> *De rerum naturâ*, p. 379. *Valpy.*

The treatment of this malady, its aggravation under certain circumstances, and the influence which it exerts over the curability of insanity, have been placed before you. The more humble task appropriately falls to me, of describing the lesions which precede or accompany this disease, or rather, which are left behind in the brain, and other organs of the body. To unfold, however, more of its pathology, I shall quit occasionally the coarse revelations of the scalpel, to discourse on the more subtle agencies which usher in this formidable malady.

Of the six epileptic patients who have died during the past fifteen months, three only exhibited special peculiarities. In all there was great congestion of the encephalon, but with this exception there was no lesion or deformity either in the brain, or the base of the skull, which is not common to chronic cases of mental derangement apart from epilepsy. I shall limit my descriptive details, therefore, to these three cases. One of them, J. G., possessed considerable interest, and she was pointed out to the gentlemen who formed the class of last year, because at the onset of her fits, she began turning round and round with great rapidity; sometimes these gyrations would be carried on for several seconds, at others, she would pass half way round, and then fall with great violence. When eight years old, she received a severe blow on the forehead, which fractured the external table of the frontal bone; the scar which remained was triangular in form, and about two inches in length, (*i. e.* from the base to the apex of the triangle.) The blow was followed immediately by epilepsy. She, however, grew up to womanhood; married, had children, and became a widow in 1837, at which time, by the combined influence of poverty, grief, and epilepsy, she became insane. She was admitted into this asylum on March 7, 1838, and was ordinarily gentle in her deportment, possessing a few hallucinations, but these were harmless in their character, and she was pleased with any attention from the officers and nurses, except for a few hours preceding the paroxysm of epilepsy, when she became morose, suspicious, jealous, and angry. At the commencement of the fit, she would rise from her seat, and pass into rapid gyrations, as before stated; when in bed, she had a lesser tendency to rotate, but even there, I have seen her roll over to her face, and from that position on to her back again. She died in the night of the 18th of December last. She was then in the 56th year of her age, and had been insane eleven years, and epileptic forty-eight years. In the autopsy, the scalp was found to be healthy. The calvarium had a deep indentation of a triangular form in the centre of the frontal bone. This was limited to the first table, as no corresponding mark was visible on the cerebral surface of the bone. The inner table was very deeply grooved by the passage of the meningeal artery; so much so, that the bone was quite thin, and diaphonous along its course. The base of the skull was irregular in form, and much contracted at the anterior part upon which the cerebral lobes rested; the "crista galli" of the ethmoid bone was large and long. The dura mater was very firmly adherent to the surface of the skull. The arachnoid membrane was opaque; the pia mater congested with blood; the Pacchionian bodies were of great size; the brain was congested with blood throughout its structure, but the congestion was most intense about the cerebellum and medulla oblongata. The lateral ventricles were devoid of fluid. The pituitary body was much atrophied. The heart was small, and its muscular structure flabby. The tricuspid valves of the right ventricle were diseased and thickened. The other viscera of the body appeared to be healthy. The other patient was J. G., whose case has been detailed to you already to some extent, when speaking of the obscurity of pulmonary disease in the insane. This patient was quite imbecile. She was admitted on the 11th of January, 1843, and had then been insane one year; her insanity was ascribed to epilepsy, and the epilepsy was produced by fright in childhood. She was in the 38th year of her age at the time of her decease, which took place from extensive disease of the lungs, at the time and in the manner already related. The calvarium was irregular in form. The dura mater was very adherent to the skull. The arachnoid membrane was opaque and milky over the cerebellum, but not elsewhere (except, indeed, around the Pacchionian glands, which I believe to be universal.) There was a large quantity of brown-coloured serum beneath the sac of the arachnoid, filling up the anfractuosities of the convolutions. The convolutions were atrophied, and widely separated from each other by the above fluids. The *form* of the encephalon was peculiar: the left lobe of the cerebrum being much larger, and three-quarters of an inch longer, than the right lobe. The vesicular neurine of both hemispheres was soft, and varying in hue from a dark brown to a pale fawn colour. The lateral ventricles contained about one ounce and a half of serum; their lining membrane was dense, gritty, and strong. The middle lobe of the cerebellum was much shrunken, and had a pale, macerated appearance, like to animal structure which had been immersed a long time in fluid. The encephalon in other parts was much congested. The pituitary gland appeared healthy. The condition of the pulmonary organs I need not repeat. The membrane of the heart, at the junction of the auricles with the ventricular openings, was inflamed on its external surface, and presented the appearance of small acupunctures in lines, or faintly resembling "psoriasis inveterata," when it attacks the angles of the mouth. The abdominal viscera were healthy. The inequality of size in the two hemispheres of the cerebrum, in this latter case, may remind some of you, perhaps, of Dr. Wigan's book, entitled, the "Duality of the Mind." The very name of the volume startled metaphysicians, and led to its neglect. It was certainly a misnomer. It advertised a paradox, if not an absurdity. Had that amiable physician given some other title to his book, it would have attracted more attention. It is a charming philosophical romance —a perfect reflex of the doctor's own mind—ingenious, sparkling, eccentric, credulous, and amusing. It is clever and analogical; and although fancy and feeling do predominate in its pages over deep thought and sound reasoning, it nevertheless contains many truths. It is moreover a honest book. It contains the genuine faith, the sincere convictions of the writer; his motive and aim in the publication are conspicuous in every page, and they are simple and pure: and, for these qualities, one may pardon much looseness of style, some illogical conclusions, and perhaps a too capacious credulity. To return to the autopsy, although I have seen another epileptic brain thus irregular in its formation, still. I have met with a third patient in whom there was this irregular development without epilepsy, (there is an exact outline of her calvarium on the table,) which enables me to state, that the above inequality had more to do with the manifestations of the mind than with the convulsions of epilepsy. The patient to whom I refer had spiculæ of bone protruding from the sides of the skull, near to the superior longitudinal sinus, on to the upper parts of the brain. The calvarium was irregular in its outline, as you perceive from the diagram before you,—the membranes were much thickened,—the vessels of the brain were atheromatous, and the cerebral hemispheres varied in size, as in the case of J. G.; yet this patient was not epileptic, but very insane; indeed, her case is so interesting, that, although it has been previously published, I cannot refrain from again alluding to it. This poor woman was blind, and suffered greatly from bronchitis. She caused great noise at night by striking at "the witches," whom she thought surrounded her. She beat the sides of her "crib-bedstead," and thus caused much noise, and bruised her own hands, in her efforts to knock down these intruders. Had she been under the care of the patrons of restraint, she would, in all probability, have had her hands muffled, and been thus left under the torturing impression that she had been overpowered by the wizards and spirits who thronged around her; under a happier arrangement, the bedstead was muffled, instead of herself, by padding it with coir, and thus she was prevented from injuring her hands, while all the noise, induced by formerly striking its boarded sides, was completely removed. I have frequently heard her hold a lengthened parley with these imaginary foes, and have seen her at other times attempt to correct her delusions; she would then carefully feel with her hands at all the points in which she imagined their presence, and not finding anything, would as attentively listen for a footfall, or other movement, (for, in conversation, she never regarded them as disembodied things, and would frequently ask me whether I did not see them pass under the bed, or out at the windows,) when on such occasions, she neither felt nor heard anything, she would say, "There's nothing—it is my fancy." If at this moment her cough commenced, she would exclaim, with an oath, "Oh, here they are again, squeezing my lungs out!"

You all remember the interesting anecdote of Nicolai of Berlin, detailed to you by Dr. Conolly, in his first lecture, furnishing as it does a fine example of a philosophic mind triumphing over the delusions of sense; but here is an opposite case, in which the mind, weakened and diseased, becomes the slave of corporeal impressions. You cannot have failed to observe, too, in your own personal experience, how powerfully the healthy senses contribute to the support of the mind when in a weak condition (to use such language). How often, in awaking from a troubled dream, do we require all the aid of the eye and the ear to convince us that it is not a reality. Again, how easy it is to yield oneself to reverie, if we exclude all the impressions conveyed to us by the senses. Hence this poor creature, shut up in a world of darkness, was constantly the prey of wild chimeras,—at one time, laughing aloud at their imaginary pranks and gambols; at another, swearing at, and cursing their insolence; in a third, struggling with terror under their inflictions,—for I have seen her black in the face, from the loaded state of the bronchial tubes, and frightened by the thought that her suffering was produced by the harpies, who were striving to strangle her. Here, then, as it appears to me, an intimate connexion between psychical phenomena and physical organization (however strange that relationship may be) is abundantly proved. An irregular, ill-shaped, constricted skull,—the meninges thickened and diseased,—small anterior cerebral lobes,—spiculæ of bone touching the superior edges of both lateral hemispheres,—the circulation through the brain interrupted by bony growths,—the blood itself impure, through an imperfect respiration,—the hemispheres unequal in size, and dissimilar in form,—a loss of the correcting power of one of the most important of the senses, and a distant physical irritant (mucus in the bronchi), lending its influence still further to disturb the already deranged seat of intelligence; these, physiologically, seem to furnish scope for the wildest delusions of the intellect, and here we perceive they existed. This, too, my friends, is one among many chronic cases in which the medical man may improve and tranquillize, even when he cannot heal; another, among hundreds, which demonstrates that an efficient medical staff is required even for chronic lunatics, since the worst features of her mental malady, its rage and its terror, were influenced by, and corresponded to, the varying states of the physical disease.

As I have before stated, this case also illustrates an important fact, namely, that certain parts of the brain may be extensively diseased without inducing epilepsy: it also stands in remarkable contrast with the following epileptic case. Thus: the patient, E—— E——, (whom many of you saw, about three weeks ago, in a semi-comatose state, and who then presented a livid appearance about the lips, cheeks, and fingers) died on the 1st instant. She had been epileptic many years, and yet, on examination after death, the brain presented no changes, except such as are common to cases of chronic mania. There were no spiculæ of bone protruding from the skull at any part; there were no cysts of any kind in the lateral ventricles, or elsewhere; in short, there was no lesion of the cerebrum or cerebellum (that I could detect) which was likely to induce epilepsy, but on looking at the base of the skull for the pituitary gland, I found it much hypertrophied, gritty, and of a brick-dust colour. The abdominal viscera were healthy, but the heart was very small, and its muscular tissue soft, flaccid, and degenerated in its structure. It weighed only five ounces and six drachms, avoirdupois. You may remember that some most distinguished anatomists have ascribed epilepsy to lesions of the little pituitary body, whose state has been just referred to; and I have frequently found this gland enlarged, and otherwise altered in epilepsy, when no other cerebral change was discernible. Its *structure*, consisting of nucleated vesicles—its blood-vessels—the manner in which it is invested by the dura-mater—and its connexion with the brain through the infundibulum, another "high-classed" body—and its being found in the brains of an extensive class of animals, induce me to attach considerable importance to its derangements, nor the less so, that it has long been regarded with respect by observers in all times. I do not believe with the ancients, that it secretes the mucus of the nostrils, more than I believe, that the pineal gland is the especial seat of the human soul; but as both these minute bodies have, for long ages, had *functions* appropriated to them, we should act unwisely to pass them slightly over in our investigations of cerebral disease. Is this body in any degree excretory in its functions? I once found this structure very much hypertrophied, in fact, of twice its normal size, in a patient who was not known, at the time of her decease, to be epileptic; but on further investigating her history, I found that she had been epileptic for many years.

Epilepsy is frequently kept in abeyance by other diseases, and is sometimes suspended for many months by a powerful appeal to the credulity, or the faith of the patient. Pulmonary consumption has been known to arrest the convulsive malady. Thus M—— A—— became phthisical three months ago, and she has not had a fit since she began to expectorate, although during one month, commencing from the 23rd day of January, she had as many as one hundred and seventeen epileptic fits. She has been closely watched during the past two months, and has not had a single paroxysm of the malady, although, previous to the above occurrence, she had four, five, and six fits daily: so true is it, as Shakspeare long ago observed, that one disease, or even one feeling, will influence the career of a second:—

"Tut, man! one fire puts out another's burning,
One pain is lessen'd by another's anguish;
Turn giddy, and be holp by backward turning;
One desperate grief cures with another's languish;
Take thou some new infection to thy eye,
And the rank poison of the old will die."

*Romeo and Juliet*, Act I., Scene 2.

To return to the necroscopic appearances. The lesions of the brain are by no means uniform in epilepsy. In one woman who died suddenly in 1847, the convolutions of the brain were strewed with a great number of knotty indurations which were caused by the cysts of true hydatids; they varied in size from that of a vetch, to a large bean, but of a corrugated and rounded figure; and one situated in the corpus striatum of the left ventricle was as large as a pigeon's egg. A few of these cysts, which contained limpid fluid, possessed within this some clear spherules, which were probably young hydatids. The corrugated hydatid membranes which had lost their fluid, contained a white grumous material, resembling an impalpably fine mortar—this grumous mass, under the microscope, was found to possess a large number of microscopic crystals having a rhombic figure. In other cases, the clinoid processes are found elongated; in three cases of epilepsy which have fallen under my notice, the brain was greatly congested, and bulged suddenly upwards and laterally, on the removal of the calvarium, so as to give the idea that it had experienced some amount of pressure from its natural covering. This condition is, perhaps, a frequent concomitant of the epileptic paroxysm, and may explain the method of cure in those rare cases, which have been relieved by tying the carotid artery, or trephining the skull. In other instances, no lesion whatever has been found in the brain at all likely to solve the phenomena exhibited during life, and we are led to suppose that the epilepsy was caused by disorganization of the kidneys—irritation in remote structures, or from a poisoned condition of the blood—instances of apoplexy from the first cause are numerous—I have seen some cases of epilepsy also ascribed to it—and the effects which follow the insertion of strychnia into the veins, is a familiar illustration of the power of a poison in the blood to induce the disease. Anything, moreover, which interrupts the circulation of blood through the medulla oblongata, the mesocephalon, and the sensory ganglia at the base of the brain may induce epilepsy. There is no known specific lesion which is pathognomonic of this disease. Congestion of the above-named structures is a frequent cause; but more frequently still, an irregular circulation, or an anæmic condition of the brain, are productive of this malady: the researches of Andral have demonstrated that anæmia, and hyperæmia excite the same train of phenomena, and that in a great number of convulsive diseases our only guide to a safe practice is the previous history of the patient. The last moments of an animal which is dying from loss of blood, resemble a fit of epilepsy. You will have observed that many of our female epileptics are far from having a ruddy, congested appearance, and I have frequently drawn the attention of gentlemen who have been with me in the epileptic wards, when a patient has been seized with a paroxysm of epilepsy, to the sudden transient pallor which pervaded the face, and the panic-look which preceded the characteristic shriek of this malady; *after* the shriek, or scream, we observe the gradual accession of the congestive stage, as the circulation becomes impeded by the universal spasm of the muscles of the head and neck. Still we should be in error, were we to regard an anæmic condition of the brain as the sole excitant of epilepsy. There are conditions of existence, and of vital phenomena, which we can never unravel. Individuals differ as widely in their susceptibilities, and in their liability to special diseases, as they do in the expression of their countenances, and in the variety of their tastes. We need go no further for illustration, than the fact, that the same kind of intelligence which blanches the cheek of one individual, paints with crimson the face of his friend. All that our present knowledge enables us to state is, that epilepsy may be produced either by somatic, or psychical causes, and that, originating in the former, it may ultimately induce intellectual disorder; while intense mental excitement may give rise to such somatic disturbance, as to produce the convulsions of epilepsy —the cases of J—— G——, and J—— G——, illustrate the first position, and instances of the latter are numerous; thus, the strong mind of that good man, John Wesley, and of his great rival in apostolic zeal, Whitfield, could remain sane, and in a comparative degree calm, while bringing before the excited imaginations of their hearers the sublime transactions of the judgment hour, the rapturous joys of heaven,—or the frightful agonies of a self-pictured hell, but not so, with many of the recipients of their doctrines; to them, such scenes, described in earnest language by a powerful fancy, assumed a present reality, and madness and epilepsy frequently sprung up like an epidemic disease. In Wesley's journal for June, 1759, I read: "I had but just spoke, when I heard a dreadful noise on the further side of the congregation, and turning thither, saw one Thomas Skinner coming forward, the most horrible human figure I ever saw —his large wig, and hair, were coal black—his face distorted beyond all description; he roared incessantly, throwing and clapping his hands together with his whole force. Several were terrified, and hastened out of his way. . . . . Not a few of the triflers grew serious, while his kindred and acquaintance were very unwilling to believe their own eyes and ears. They would fain have got him away; but he fell to the earth, crying: 'My burden, my burden. I cannot bear it.' Some of his brother scoffers were calling for horse-whips, 'till they saw him extended on his back, at full length. They then said he was dead; and indeed, the only signs of life were *the working of his breast*, and the *distortions* of his *face*, while *the veins of his neck were swelled, as if ready to burst.*" Again, in another place, after describing similar scenes, under other preachers, the following observations are recorded: "Some said these were purely natural effects; the people fainted away only, because of the heat and closeness of the rooms," and others were sure "it was all a cheat; they might help it, if they would. Else, why were these things only in their private societies? Why were they not done in the face of the sun?—to-day I was enforcing these words (here follows a text) not in a close room, neither in private, but in the open air, and before more than 2000 witnesses. One, and another, and another, was struck to the earth exceedingly trembling. . . . One person dropped down close to me who was a strong assertor of the contrary doctrine. While he stood astonished at the sight, a little boy near him was seized in the same manner. A young man who stood up behind, fixed his eyes on him, and sank down himself, as one dead; but soon began to roar out, and beat himself against the ground, so that six men could scarcely hold him."

Similar scenes are frequently recorded in other parts of John Wesley's Journal.

*Charles Wesley* arrested the spread of this mental epidemic among his congregation, by distinctly telling them from the pulpit, that he thought nothing better of them for these convulsive attacks, and by directing his porters to take the persons thus afflicted to the outside of the chapel, *and to leave them there.* After this, he states, "his porters had nothing to do." (Southey's Life of Wesley.) Lord Byron presented a remarkable instance of the power of psychical influences in inducing this malady. Thus, at the 77th page of the third volume of Moore's Life of Lord Byron, we read,—"Such effect had the passionate energy of Kean's acting on his mind, that once, in seeing him play Sir Giles Overreach, he was so affected as to be seized with a sort of convulsive fit; and we still find him, some years after, in Italy, when the representation of Alfiéri's tragedy of Mirza had agitated him in the same violent manner, comparing the two instances as the only ones in his life when anything under reality had been able to move him so powerfully." Some of the epidemics of the Middle Ages assumed the convulsive form under intense religious excitement; and it has been calculated that, even in England, on the introduction of Methodism, upwards of 4000 persons were, within a very short period, affected with convulsive diseases. Insanity and epilepsy depend for their manifestation on special and separate parts of the encephalon, and hence, throughout my observations, in order to keep its pathology distinct, I have been referring to the latter malady as though it had been idiopathic, or apart from the former. Dr. Conolly has already detailed to you, that when it supervenes upon, or even when it has preceded mental derangement, it renders the insanity well-nigh incurable; and the prognosis is thus unfavourable, from the supposition that the disease has extended through a large portion of the encephalon—has reached from the periphery of the brain to the great ganglia at its base—or has spread from these to the surface of the hemispheres. I need not, however, tell you that epilepsy may exist without *necessarily* involving the intellect. The "foremost man of all this world,"—he whom Brutus slew—was an epileptic; and many Greek writers maintain, that the conqueror in the battle-fields of Beder and Ohud—the great iconoclast and prophet of Arabia—was subject to paroxysms of this fearful disease; as is the late distinguished exile of Gaeta, but a few months ago the most loved, as he was the most liberal and the most enlightened of a long line of illustrious pontiffs.

There is one important fact in connexion with the epileptic patients, who have died during the period we have been reviewing, which I have not yet alluded to,—the deaths of two of them were quite sudden. One died while taking a bath, a second while having her breakfast. In both instances it is certain that there was not a series of epileptic paroxysms. Now, had they died in bed, with the face downwards,—and in three-fourths of those who die the face is prone,—their deaths may have given rise to some doubts as to whether their end had been accelerated by the position which the face had assumed in reference to the bedclothes. Here, however, no such cause could be assigned for the fatal result, the death in each case being caused by apoplexy,—*i. e.*, by extravasation of blood upon the brain, or by a fatal congestion of its vessels. Death ensues in epilepsy from various causes. I have given to this subject great attention, and my belief now is, that the most frequent cause of death is venous congestion of the brain, or an effusion of blood, constituting "apoplexy."

2nd. From asphyxia, the result of congestion or pressure upon the mesocephalon, and the upper part of the spinal chord,—the "respiratory tract" of Bell.

3rd. From spasm of the glottis, continuing long enough to induce such changes in the blood as paralyze the action of the heart, or end in fatal coma.

4th. I think it is *possible* that during the epileptic paroxysm, the face being pressed on the pillow, and that part of the sheet opposite the mouth being saturated with saliva, that suffocation may ensue from a mechanical exclusion of the air by the *wetted* sheet, and the pressure of the head upon it;—*i. e.* I believe a fatal termination may ensue under such circumstances, which would not arise if the face was freely exposed to the atmospheric air: first, because the pre-existing congestion of the brain and spinal chord has blunted the respiratory sense, and thus reduced the *energy* of the inspiratory act; next, the inspiratory act, in the absence of all consciousness on the part of the patient, with the nose closed by the pillow, even if excited, may draw up the *wetted* sheet into the mouth, and thus, without excluding *all air*, may admit it in quantities too small for the continuance of life; and in these rare cases, the usual characteristics of suffocation would be more conspicuous than in those where *all* air had been instantaneously shut off from the patient.

It is this sudden and unexpected demise of many epileptic patients which imparts to this disease additional importance; and which, as you have already heard, formed one of the greatest barriers to the introduction of the Non-Restraint System. It was said to be necessary to fasten the epileptics, to keep them on their backs, lest they should be suffocated during the night in a paroxysm of their malady. It is one of those arguments which, being founded in error, must totter and fall, as fact after fact reveals itself. It is one of those objections which gives me very little concern. Statistics will destroy and erase it. Given, that a patient, one in five hundred, rolls on to her face, and becomes suffocated by that circumstance,—where is the justifiable "restraint" which will prevent it? Did not as many patients die in this asylum from supposed suffocation, as now, at a time when they were fastened by the hand every night to prevent it? There are no records. But those who knew the asylum well, then and now, admit the fact. If such be the fact, then all argument is at an end. It is enough, that a system which produces greater comfort, greater happiness, and an equal number of cures, should be *as safe* as the more harsh plan, which it has displaced; being this, its adoption is not only justifiable, but absolutely necessary; nay, it cannot be withheld, except in violation of some of the most sacred duties which man owes to man. There is no need of an elaborate, controversial disquisition on the physiology of the disease, to demonstrate the inutility of such fastenings, or to justify the absence of restraint. It is more than justified by the above-named facts; and this, by an argument which is understood by the wise, and the simple, by the philosopher and the child, and by the unlearned, as well as by the professional man.

---

